# Comprehensive mixed reality surgical navigation system for liver surgery

**DOI:** 10.1117/1.JMI.12.5.055001

**Published:** 2025-10-06

**Authors:** Bowen Xiang, Jon S. Heiselman, Michael I. Miga

**Affiliations:** a Vanderbilt University, Department of Biomedical Engineering, Nashville, Tennessee, United States; b Vanderbilt Institute for Surgery and Engineering, Nashville, Tennessee, United States; c Vanderbilt University Medical Center, Department of Neurological Surgery, Nashville, Tennessee, United States; d Vanderbilt University Medical Center, Department of Radiology and Radiological Sciences, Nashville, Tennessee, United States; e Vanderbilt University Medical Center, Department of Otolaryngology – Head and Neck Surgery, Nashville, Tennessee, United States

**Keywords:** surgical navigation, liver surgery, image-guided surgery, mixed reality, augmented reality, deformation correction, tracking

## Abstract

**Purpose:**

Intraoperative liver deformation and the need to glance repeatedly between the operative field and a remote monitor undermine the precision and workflow of image-guided liver surgery. Existing mixed reality (MR) prototypes address only isolated aspects of this challenge and lack quantitative validation in deformable anatomy.

**Approach:**

We introduce a fully self-contained MR navigation system for liver surgery that runs on a MR headset and bridges this clinical gap by (1) stabilizing holographic content with an external retro-reflective reference tool that defines a fixed world origin, (2) tracking instruments and surface points in real time with the headset’s depth camera, and (3) compensating soft-tissue deformation through a weighted ICP + linearized iterative boundary reconstruction pipeline. A lightweight server–client architecture streams deformation-corrected 3D models to the headset and enables hands-free control via voice commands.

**Results:**

Validation on a multistate liver-phantom protocol demonstrated that the reference tool reduced mean hologram drift from 4.0±1.2  mm to 1.1±0.3  mm and improved tracking accuracy from 3.6±1.3 to 2.3±0.8  mm. Across five simulated deformation states, nonrigid registration lowered surface target registration error from 7.4±4.8 to 3.0±2.7  mm—an average 57% error reduction—yielding sub-4 mm guidance accuracy.

**Conclusions:**

By unifying stable MR visualization, tool tracking, and biomechanical deformation correction in a single headset, the proposed platform eliminates monitor-related context switching and restores spatial fidelity lost to liver motion. The device-agnostic framework is extendable to open approaches and potentially laparoscopic workflows and other soft-tissue interventions, marking a significant step toward MR-enabled surgical navigation.

## 1 Introduction

 Hepatic tumors constitute a significant healthcare challenge both nationally and internationally. Primary liver cancer affected ∼41,630 individuals in the United States in 2024, resulting in 29,840 fatalities.^1^ Globally, the incidence and mortality rates exceed these figures by more than 20-fold.^1^ Notably, primary liver cancer incidence has shown a threefold increase since 1980.^1^ Although hepatocellular carcinoma represents an important subset of primary liver malignancies, metastatic disease accounts for ∼76% of liver tumors across treatment facilities. Among these metastases, ∼75% originate from colorectal carcinoma. Consequently, metastatic colorectal carcinoma represents the most frequently treated hepatic malignancy, with hepatic metastasectomy offering potential long-term survival for appropriately selected patients. Given that over 150,000 new colorectal cancer diagnoses are anticipated this year,^1^ with ∼75%^2^ subsequently developing hepatic metastases, the clinical burden and therapeutic imperative of liver-localized malignancy remain substantial.

In evaluating curative interventions for hepatic malignancies, liver transplantation represents the gold standard therapeutic approach, although its widespread application remains constrained by organ availability. Hepatic resection constitutes the next most efficacious treatment modality, necessitating meticulous preoperative planning to precisely localize subsurface tumors and critical vascular structures. For patients with unresectable disease, locoregional therapies—particularly thermal ablation—serve as important bridging interventions preceding definitive resection or transplantation.^3^ Regardless of the selected treatment modality, comprehensive preoperative planning is imperative to ensure oncologic margins, optimize functional hepatic reserve, and mitigate surgical complications, including hemorrhage and biliary injury. A significant technical challenge in hepatic surgery involves intraoperative organ deformation resulting from surgical manipulation, including perihepatic packing in open procedures, pneumoperitoneum in laparoscopic approaches, retraction maneuvers, and mobilization from supporting ligamentous structures.^4^ These deformations substantially diminish the fidelity of preoperative imaging data when applied intraoperatively. Quantitative analyses have demonstrated significant morphologic discrepancies between preoperative and intraoperative hepatic configurations in both open and minimally invasive approaches.^5^ Advancements in biomechanical modeling, specifically the linearized iterative boundary reconstruction (LIBR) methodology, have demonstrated substantial promise in compensating for these intraoperative deformations.^4^

Image-guided surgery (IGS) systems provide significant clinical utility through their ability to spatially correlate preoperative data with intraoperative anatomy. Among commercially available platforms for hepatic applications, CAS-One^®^ IR (CASCINATION AG, Bern, Switzerland) represents a state-of-the-art implementation. Nevertheless, conventional IGS platforms exhibit inherent limitations in their visualization paradigm. Specifically, the navigational display is typically positioned at a considerable distance from the surgical field, necessitating that clinicians repeatedly redirect their attention between the operative site and the guidance system. This continuous context-switching imposes a substantial cognitive burden as surgeons must mentally transform and integrate spatial information between disparate reference frames.^6^ Mixed reality (MR) technology presents a promising solution to this fundamental limitation. MR environments facilitate bidirectional interaction between virtual and physical elements, enabling contextually responsive digital objects that dynamically integrate with the surgical field. This technological approach establishes a unified perceptual framework where digital and physical components coexist and interact synchronously.^7^

Currently, MR-based navigation systems have predominantly been implemented in surgical disciplines characterized by rigid anatomical structures, including dental implantology,^8^ parotid tumor surgery,^9^ and cranial and spinal surgery.^10^^–^^12^ The biomechanical stability of these structures facilitates robust registration and visualization of holographic models, enabling precise surgical planning and intraoperative guidance.^13^ Current research has expanded these applications to include robotic-assisted procedures, where MR facilitates teleoperation and dynamic control of robotic surgical systems.^14^ Limited investigations have explored MR implementation in deformable anatomical contexts, such as brachial plexus surgery, where structural relationships vary with positional changes.^15^ In addition, chest wall procedures have incorporated MR visualization, though these applications have primarily treated the target anatomy as rigid, with authors acknowledging the future necessity of addressing tissue deformation dynamics.^16^ Despite these developments, the literature currently lacks a comprehensive MR-based navigation system specifically designed for hepatic surgery with quantitative accuracy assessment—a critical gap addressed by the proposed system.

Addressing the clinical gap in liver surgery requires the integration of advanced imaging modalities, real-time data fusion, and intuitive visualization technologies. The combination of preoperative CT/MRI data with intraoperative organ surface information, augmented by sophisticated soft tissue deformation correction methods and enhanced visualization through augmented reality, offers significant potential for improving the accuracy and reliability of liver tumor localization. To realize this potential, this paper presents the development, validation, and preliminary evaluation of a surgical navigation system utilizing an MR headset, tailored specifically for liver surgery.

This paper presents several significant contributions in MR-based surgical navigation for liver surgery. First, we conduct a comprehensive evaluation of HoloLens^®^ 2’s built-in simultaneous localization and mapping (SLAM) functionality, demonstrating its limitations for clinical applications and introducing an external reference target that significantly improves hologram stability under various surgical conditions. Second, we rigorously assess the tracking accuracy of the MR headset’s depth camera both with and without the external reference, establishing quantitative metrics that validate our approach. Third and most importantly, we develop a complete end-to-end self-contained system that addresses critical clinical gaps in liver surgery by integrating three essential components: precision tool tracking, biomechanical model-based deformation correction, and intuitive mixed reality visualization. This integration enables surgeons to visualize subsurface tumors and vascular structures with enhanced stability, intuitive interaction, and improved accuracy. Finally, we provide preliminary validation through surface target experiments that demonstrate significant improvements in target registration error (TRE) when using our nonrigid registration methods (NRMs). This holistic approach represents a substantial advancement in surgical navigation technology, potentially improving surgical precision and patient outcomes in liver surgery. A preliminary version of this system was previously presented as a feasibility study in the *Proceedings of SPIE Medical Imaging 2025*, demonstrating initial validation on a liver phantom.^17^ The current work significantly extends this prior study by introducing a stable external reference tool and preliminary surface targets-based evaluations.

## 2 Method

### 2.1 Comprehensive MR-Based Guidance System

**Fig. 1:**
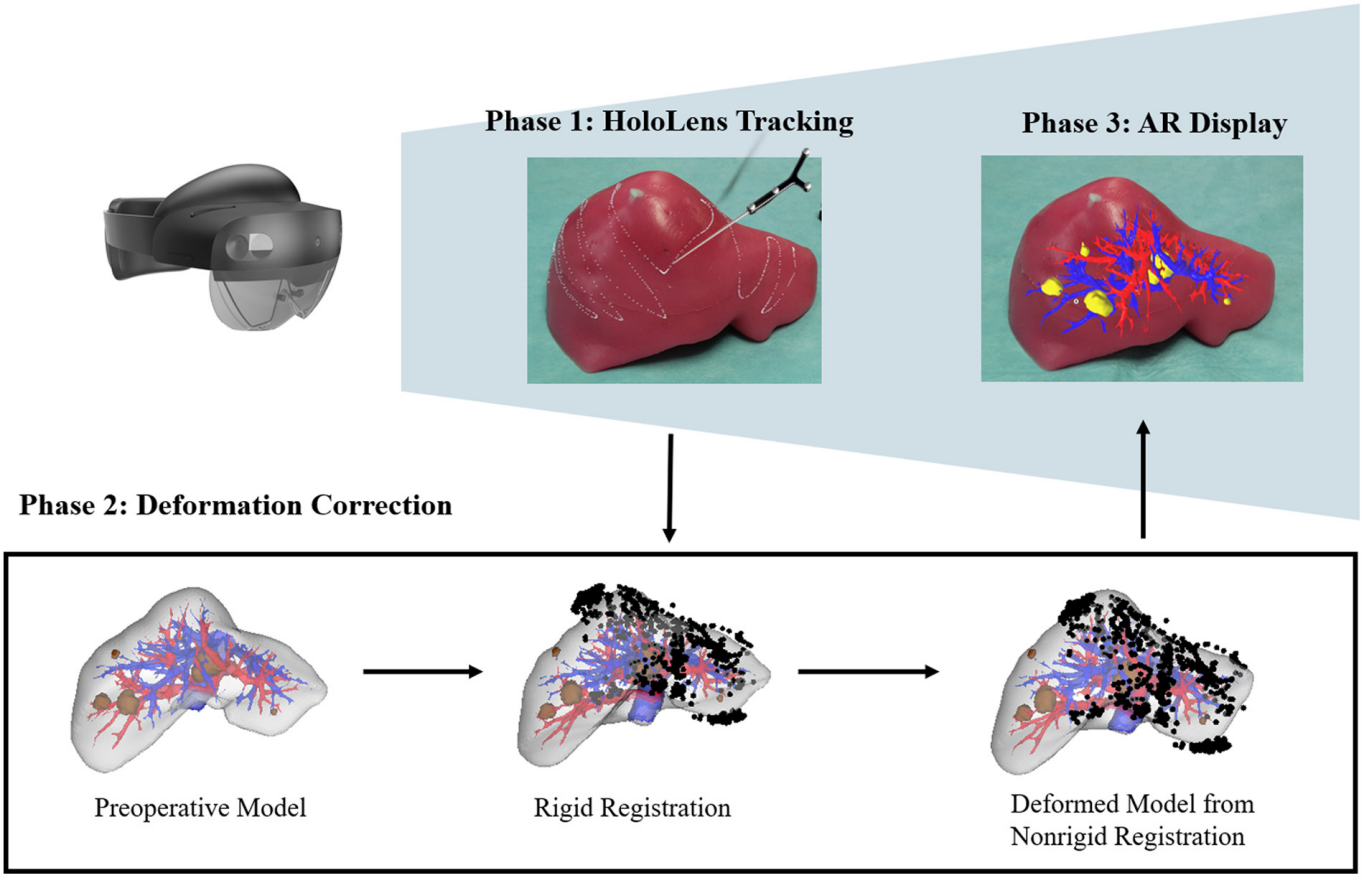
System overview showing the three main phases of the surgical navigation system: HoloLens^®^ 2 tracking for surface data acquisition (phase 1), deformation correction using rigid and NRMs (phase 2), and AR display of the deformation-corrected model (phase 3).

To develop an advanced surgical navigation system for liver surgery utilizing MR, we employed a robust approach comprising three primary stages: tool tracking, deformation correction, and dynamic 3D model display in a mixed reality environment. The system utilizes the Microsoft HoloLens^®^ 2 (Redmond, WA) as its chosen MR headset. HoloLens^®^ 2 incorporates a depth camera operating in articulated hand tracking (AHAT) mode, which provides wide-field infrared depth and reflectivity data at 45 fps. Optimized for near-range sensing up to 1 m, this modality enables reliable real-time detection of retro-reflective markers and surgical tools. In the clinical workflow, the patient undergoes preoperative imaging to acquire data for segmentation of the liver, internal vasculature, and tumors, enabling the creation of detailed preoperative 3D models. Following conventional surgical manipulation, which induces liver deformation, intraoperative liver surface data are collected using the AHAT camera of the HoloLens^®^ 2 and a tracked stylus. These intraoperative surface data are then integrated with the preoperative 3D models to generate deformation-corrected models that best align with the observed intraoperative anatomy. The deformed virtual models are then projected within the patient in the appropriate physical location, thus providing a visualization of predicted subsurface anatomical structures and occult liver metastases. This approach aims to enhance intraoperative decision-making by accurately representing the anatomical locations of key subsurface features. The overall spatial relationships and system components are illustrated in Fig. 1. The tool tracking framework, pivotal to our system, was designed to accurately detect and localize surgical instruments using the AHAT camera of the HoloLens^®^ 2. This involved detecting retro-reflective markers, defining the geometrical properties of the tools, and implementing tool recognition and localization algorithms.^18^ Subsequently, deformation correction was achieved through a combination of rigid and NRMs.^4^^,^^19^ These methods ensured precise alignment of preoperative and intraoperative liver data, accounting for both rigid body movements and soft tissue deformations. Finally, our system incorporated a server-client architecture to facilitate the real-time transfer and display of 3D models in the HoloLens^®^ 2 and enable seamless visualization. The details of the system will be elaborated in the following sections.

### 2.2 Hologram Stability for MR Device

Building upon Ref. 20, it has been observed that the reliance on the HoloLens^®^ 2’s built-in SLAM functionality can result in hologram displacement. This instability is particularly problematic for clinical applications, where precise spatial consistency is critical, especially during surgical procedures. To address this, we conducted a comprehensive evaluation of the HoloLens^®^ 2 under similar conditions to assess the extent of hologram drift and determine whether the integration of an external reference tool is necessary to ensure stable hologram alignment.

**Fig. 2:**
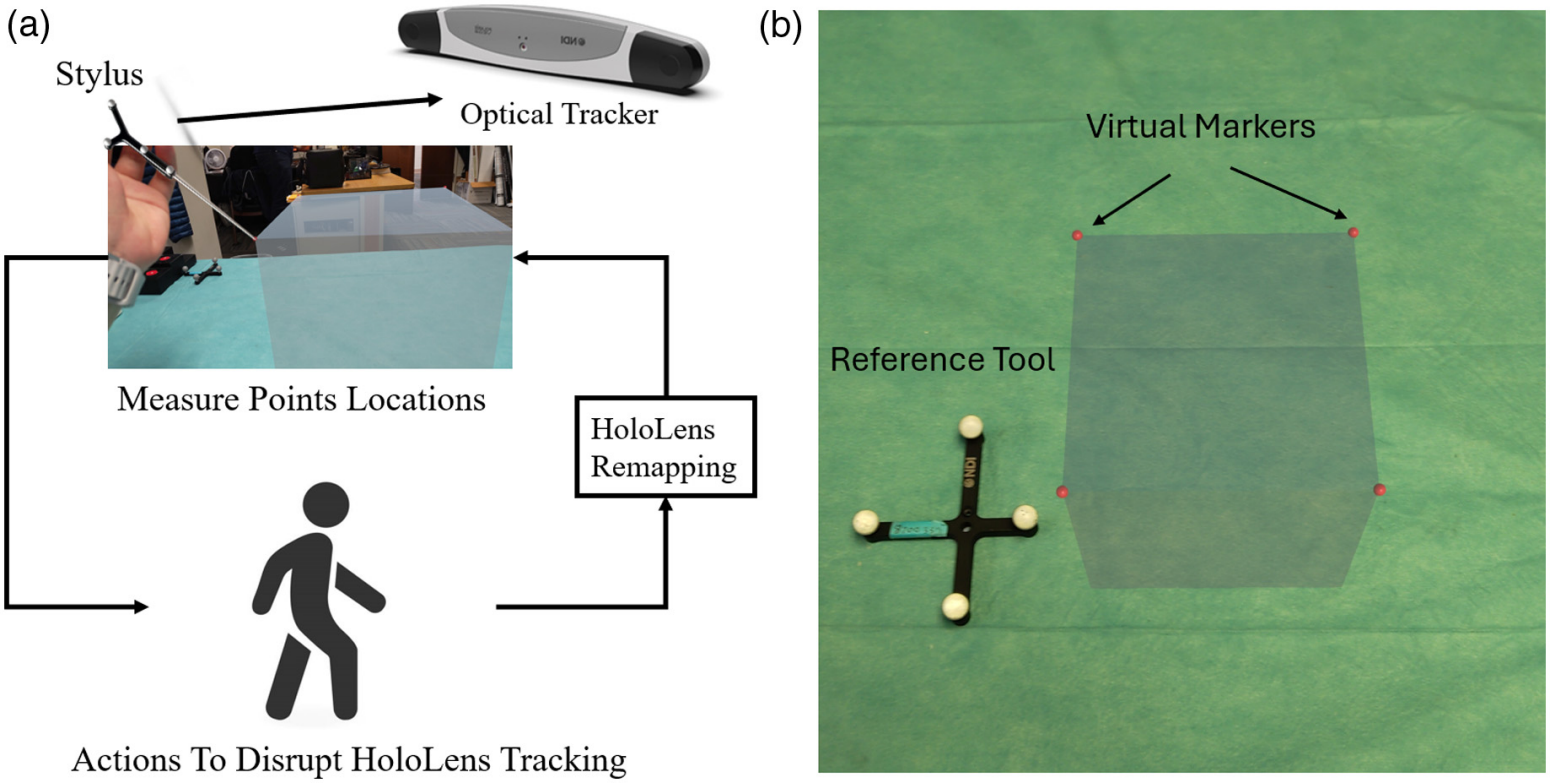
Experimental setup for evaluating hologram stability in HoloLens^®^ 2 under various perturbation conditions. (a) The setup includes a holographic cube (virtual cube) digitized using a stylus tracked by the Polaris Vega system. Conditions tested include walking, rapid head movements, sensor occlusion, and environmental changes. (b) The setup with the external reference tool.

The experimental protocol was designed to quantitatively evaluate the spatial stability of holograms rendered by the Microsoft HoloLens^®^ 2 under conditions that could potentially disrupt its SLAM functionality. The evaluation setup consisted of a HoloLens^®^ 2 device, a Polaris Vega optical tracking system (Northern Digital Inc., Waterloo, Ontario), and a passive marker-equipped stylus for spatial digitization. Prior to testing, the HoloLens^®^ 2 was calibrated to the operator’s interpupillary distance using the device’s built-in eye tracking and calibration procedure. A standardized holographic cube with a square base was generated using the Mixed Reality Toolkit (MRTK) and anchored 10 cm above a planar surface within the operator’s field of view. To measure hologram drift, the operator digitized the four top vertices of the holographic cube in a consistent clockwise sequence using the Polaris Vega tracked stylus, with points recorded via the PLUS toolkit integrated with 3D Slicer software. The experiment is shown in Fig. 2.

Four distinct experimental conditions were evaluated: (1) walking—the operator walked 7 m away from and back to the hologram, (2) rapid head movement—the operator performed sudden lateral head movements, (3) sensor occlusion—the device’s tracking sensors were temporarily covered until tracking was lost and subsequently regained, and (4) environmental change—an object was inserted into the hologram’s space for 60 s, forcing spatial mesh updates. For each condition, 40 trials were conducted, with vertex positions digitized before and after the disrupting action. The magnitude of hologram drift was calculated as the three-dimensional displacement vector between corresponding vertex positions. The Polaris system provided a tracking accuracy of ∼0.64  mm RMS. Potential sources of experimental error included stylus calibration uncertainty, operator hand tremor during digitization, and systematic bias in point selection. To minimize these effects, the operator was allowed to adjust their viewing angle for optimal vertex visualization, and consistent digitization techniques were maintained throughout all trials. This protocol enables quantitative assessment of the HoloLens^®^ 2’s spatial anchoring capabilities under controlled perturbations, providing insights into its potential suitability for precision-critical mixed reality applications.

The past experience with HoloLens^®^ 2 showed that holographic objects displayed often experienced a spatial drift, with the displacement occurring in varying directions across repeated trials of the same perturbation. This variability highlights a critical limitation: the instability of the holograms renders the device unsuitable for clinical applications requiring high precision, such as intraoperative guidance. Such unpredictability in hologram placement compromises the accuracy of surgical navigation and could hinder its adoption in clinical workflows. To address this limitation, an external reference tool equipped with retro-reflective markers was integrated into the system, as described in Sec. 2.3. This tool serves as a stable world origin, enabling HoloLens^®^ 2 to maintain consistent spatial alignment of holograms regardless of environmental or user-induced disruptions. The integration leverages HoloLens^®^ 2’s tracking capabilities to ensure that virtual models remain anchored within the physical workspace. Subsequent experiments were conducted to evaluate hologram stability with the reference tool in place. The results demonstrated a significant improvement in stability, with minimal drift and consistent positioning of virtual objects. This marked contrast between the stability outcomes with and without the reference tool underscores its essential role in enhancing the reliability of the system for precision-critical tasks. These findings provide evidence of the necessity for an external reference tool to ensure the spatial fidelity of holograms in mixed reality environments. By resolving the instability issue, the proposed solution not only addresses a major challenge but also enhances the feasibility of adopting HoloLens^®^ 2-based systems for surgical navigation. This development represents a key step toward bridging the gap between augmented reality technology and its practical application in complex clinical procedures, paving the way for further advancements in precision surgery and a true mixed reality guidance capability.

### 2.3 MR Headset Tracking

**Fig. 3:**
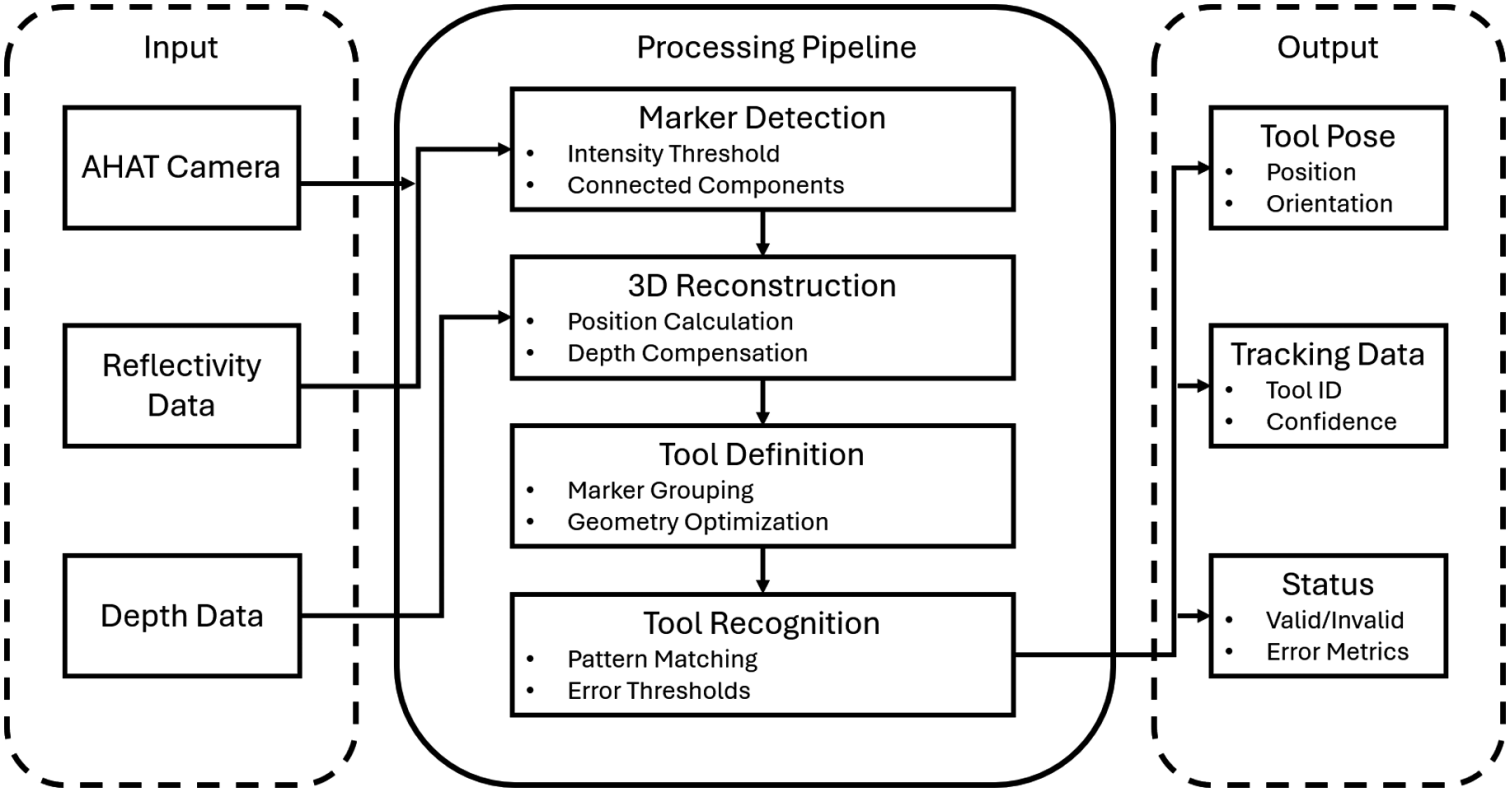
Overview of the HoloLens^®^ 2 tracking pipeline showing the system’s three main stages: marker detection using AHAT camera and reflectivity data, 3D reconstruction and tool definition through marker grouping and geometry optimization, and tool recognition for determining position and orientation in real time.

In our system, we utilized a robust framework for tool tracking using the AHAT camera in MR HMDs, specifically the HoloLens^®^ 2.^18^ This framework is structured into three primary stages: three-dimensional marker center detection, tool definition, and tool recognition and localization, as shown in Fig. 3. The initial step involves detecting the 3D positions of individual retro-reflective markers. These markers exhibit extremely high-intensity values in the reflectivity images from the AHAT camera. To identify these markers, an intensity-based threshold is applied to separate them from other objects in the scene. The reflectivity images are represented in a 16-bit unsigned integer format, where retro-reflective markers have values significantly higher than the surrounding environment, often exceeding 2000 at their centers. A connected component detection algorithm is then employed to isolate individual markers, taking into account environmental noise such as reflections from flat surfaces or random noise. An additional threshold on the connected component area helps in accurately extracting the individual markers. Using the depth information from the AHAT camera, the 3D position of each marker is calculated by back-projecting the central pixel of each marker into a unit plane using the intrinsic parameters of the camera. Once the markers are detected, the next step is tool definition. This involves extracting the 3D distribution of the markers from a single frame to generate the geometrical properties of the tool. An optimization method is used to calculate the shape of the rigid tool using the positions of the markers extracted from each frame. This step involves minimizing the root mean square error between the observed positions of the markers in different frames to refine the tool’s configuration. The final tool definition is generated by removing the mean translation of the set of markers, centering the tool at the geometric center of all markers. The final stage is tool recognition and localization. This involves identifying multiple tools from a single AHAT frame by estimating the Euclidean distance between detected markers and predefined tool shapes. A depth-first graph searching algorithm is used to find subsets of detected markers that match the shape of the defined tools. A loss function, considering the corresponding lengths of the tool, quantitatively depicts the similarity between the detected marker configuration and the tool definition. To ensure the accuracy of tool recognition, two thresholds are applied: one for identifying mismatches between corresponding sides of the tool and another for the overall tool shape. These thresholds are calculated based on the standard error of the detected marker positions, which varies with depth. This approach allows the framework to accurately recognize and localize multiple tools simultaneously in the scene.^18^

To customize the tracking method for our system, our tracking framework incorporates an external reference tool to act as the world origin due to the necessity of a stable holographic reference. This tool, outfitted with retro-reflective markers, is critical for maintaining spatial stability, addressing the inherent limitations of the HoloLens^®^ 2’s built-in SLAM functionality. The reference tool anchors the mixed reality environment to a fixed coordinate system, ensuring consistent alignment of virtual objects with the physical workspace even under dynamic conditions. To implement this approach, the system simultaneously tracks two tools: a stylus and the external reference tool. The stylus functions as a data acquisition instrument, capturing precise spatial information required for tasks such as surface measurements and intraoperative point collection. Furthermore, we assigned a tip transform in Unity to represent the stylus tip. This allows us to use the stylus to measure surface data accurately. Meanwhile, the reference tool serves as the world origin, providing a stable basis for all positional data and virtual model overlays. By tying all measurements and visualizations to this external anchor, the system ensures a robust and unified coordinate framework. This dual-tool tracking approach not only enhances hologram stability but also forms the backbone of the entire navigation system. It enables seamless integration of tracking, data collection, and visualization, facilitating precise and reliable augmented reality applications in surgical workflows. In addition, we implemented voice commands, “record points” and “stop recording points,” to facilitate the recording process. To integrate HoloLens^®^ 2 tracking with our next steps, we established a UDP connection, enabling the transmission of recorded tracking data to a PC. This setup enhances the overall efficiency of our data collection process.

### 2.4 Tracking Assessment

To evaluate the effectiveness of the external reference tool in improving tracking accuracy, we conducted a comparative analysis of the HoloLens^®^ 2 tracking performance with and without the reference tool. As noted earlier, the inherent limitations of the HoloLens^®^ 2’s SLAM functionality can result in positional drift during measurements, compromising the precision required for clinical applications. This assessment aims to quantify the extent of improvement introduced by incorporating the external reference tool. The validation of HoloLens^®^ 2 tracking precision and accuracy was performed using a machined multi-level block object designed with nine platforms of varying heights. Each platform was marked with a self-adhesive fiducial marker at its center, and the precise geometric positions of these marker centers were predetermined. For this study, a total of 10 datasets were collected, encompassing 90 individual data points. The experimental process involved capturing the point cloud corresponding to each platform center using the tip of the stylus tracked by HoloLens^®^ 2. The center of each point cloud was then identified, followed by point registration to align these identified centers with the known geometric centers of the platforms. In this context, “precision” refers to how consistently the measured points are distributed around each platform’s center. It reflects the spread of the data and is indicative of the system’s repeatability. Conversely, “accuracy” is defined as the fiducial registration error, which represents the distance between the registered centers of the measured data and the known geometric centers of the platforms after alignment. Both metrics are crucial for assessing the reliability of the tracking system as they provide complementary insights into its performance. This methodology enabled a comprehensive characterization of the tracking system’s precision and accuracy. Unlike previous studies that primarily assessed HoloLens^®^ 2’s ability to track static reflective markers, this work focused on evaluating its accuracy in tracking a moving tool.

### 2.5 Deformation Correction

The current rigid registration method (RRM) protocol consists of two sequential steps. The first step employs point-based registration (PBR), leveraging anatomical landmarks that are identifiable in both preoperative imaging data and intraoperative presentations to generate an initial pose estimation. The second step involves a surface-based registration, implemented through a modified iterative closest point (ICP) algorithm, which aligns the preoperative liver surface with the intraoperative point cloud collected by the HoloLens-tracked stylus.^19^ To enhance the robustness of this process, the weighted ICP (wICP) approach is employed, incorporating prominent anatomical liver features to refine the initial pose estimation. Specifically, a weighting scheme assigns greater significance to salient features, such as the falciform ligament, the left inferior ridge, and the right inferior ridge, which are consistently identifiable in both datasets.

Building upon this rigid alignment as an initial pose, an NRM is applied to account for soft tissue deformations. The LIBR approach extends beyond rigid registration by incorporating a biomechanical model that accounts for complex soft tissue deformations.^4^ During preoperative planning, the method first establishes a basis for potential deformations by systematically distributing control points across the liver surface. These control points are then perturbed along orthogonal directions to generate a linear basis consisting of fundamental elastic responses to spatially localized deformation boundary conditions within the tissue model. During the intraoperative phase, the method reconstructs the actual deformation state by estimating boundary condition parameters simultaneously with the rigid transformation parameters through an optimization framework that leverages closed-form gradient functions. This framework minimizes a loss function consisting of two key components: the discrepancy between the observed intraoperative data and the deformed model predictions, and the overall strain energy of the system

$$\Omega (\beta )=\underset{F}{\sum }\frac{w_{F}}{N_{F}}\overset{N_{F}}{\underset{i=1}{\sum }}f_{i}^{2}+w_{E}f_{E}^{2},$$ (1)

where $f_{i}$ denotes the error between the deformed organ model and an intraoperative data point within an intraoperatively collected point cloud for feature F of size $N_{F}$, $w_{F}$ is the weight of the feature, $f_{E}$ is the average strain energy of the deformation state, and $w_{E}$ is a regularizing strain energy weight that controls the deformability of the registration. This dual optimization ensures that the resulting deformation not only matches the available intraoperative measurements but also maintains physically plausible tissue behavior governed by the biomechanical model constraints. The outcome is a volumetrically deformation-corrected model that accurately represents the intraoperative state of the liver, capturing both surface alignment and internal structural relationships. These deformation-corrected models are subsequently projected as a virtual anatomical representation within the patient’s liver in true physical space, representing an interactive mixed reality, all facilitated by the MR headset, and enhancing intraoperative visualization and localization.

### 2.6 Mixed Reality Display

**Fig. 4:**
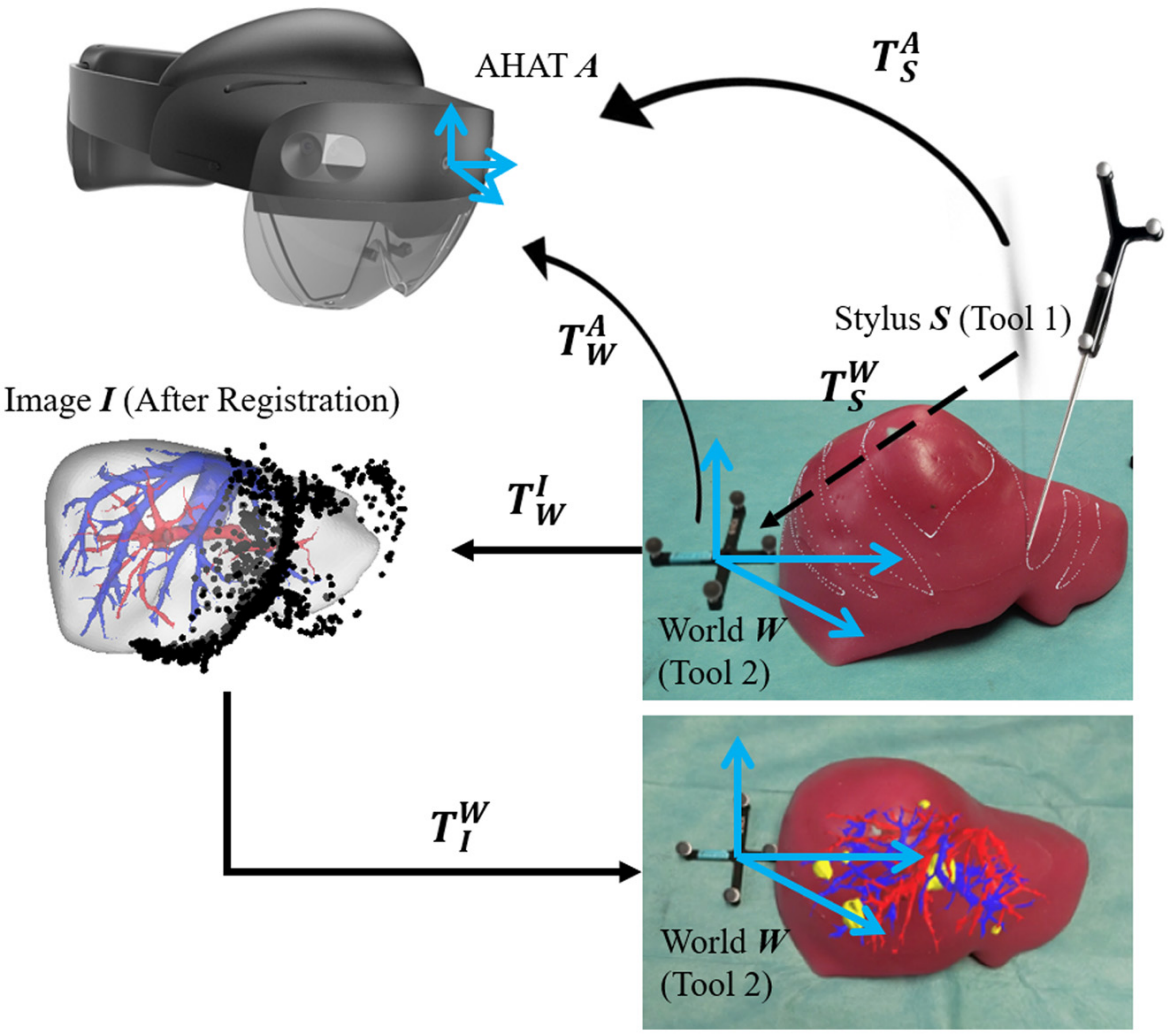
Transformation relationship for the system with reference tool. $T_{S}^{A}$ is the position of the stylus relative to the AHAT coordinate system. $T_{W}^{A}$ is the position of the reference tool (world frame) relative to the AHAT coordinate system. $T_{S}^{W}$ is the position of stylus relative to the reference tool calculated by $T_{S}^{A}$ and $T_{W}^{A}$. $T_{W}^{I}$ is the transformation matrix from the world frame to the image frame generated by the registration process. $T_{I}^{W}$ is the transformation matrix from image frame to world frame and is used to display the models at the real-world location.

In this step, HoloLens tracking serves as the world origin for the displayed virtual models. The transformation relationship between each component of the system with the external reference is shown in Fig. 4. This system features a server-client architecture designed to facilitate the efficient transfer and display of 3D models in a mixed reality environment using HoloLens^®^ 2. The server, implemented in Python, manages the distribution of model files and transformation data, categorizing them into rigid and nonrigid types, each stored in separate directories. Upon establishing a client connection, the server transmits a batch of files, including the 3D models and a transformation matrix. The client, developed in C# for Unity, receives and processes these files, rendering the models in the HoloLens^®^ 2 view. It maintains a persistent connection with the server, handling potential disconnections and automatically attempting to reconnect. Once the model data are received, the client saves the files locally, reads the transformation matrix, and loads the 3D models, scaling, transforming, and positioning them correctly. The external reference tool is set as the world origin, ensuring that the display scene and tracking scene share the unified coordinate system.

In implementing the HoloLens display system, careful attention must be given to the coordinate system conventions to ensure proper alignment and visualization of virtual models. The HoloLens ecosystem involves multiple transitions between coordinate systems due to the differing conventions used by its components, such as the AHAT camera, the HoloLens built-in world space, unity, and external visualization tools such as ParaView. These transitions require deliberate handling to avoid inconsistencies that could affect the accuracy and usability of the system. The AHAT camera operates using a left-handed coordinate system. However, when data are processed by the HoloLens built-in world space via the Mixed Reality Toolkit (MRTK), the coordinate system is automatically converted to a right-handed convention. This initial switch ensures compatibility with the HoloLens environment but introduces a disparity with some external tools and libraries. For example, in the custom C++ library used in this work,^18^ another transformation is applied to revert the data back to a left-handed system, aligning with the intrinsic requirements of our algorithms. For visualization purposes in ParaView, the data must again be converted to a right-handed system to maintain compatibility with its display conventions. Following registration, an additional transformation is required when preparing the data for Unity. In this step, the data are flipped back to a left-handed system to ensure accurate rendering within unity’s development environment. Finally, during deployment to the HoloLens via OpenXR settings, unity applies another transformation, returning the data to a right-handed system, which aligns with the HoloLens display conventions. This intricate sequence of coordinate system transformations underscores the complexity of managing spatial data in mixed reality applications. By systematically addressing these transitions, our implementation ensures that the displayed virtual models maintain spatial fidelity, aligning accurately with both the physical environment and user expectations, enabling a seamless mixed reality experience.

To enhance visual distinction, each model is assigned a unique color from a predefined palette. The client organizes the models into two containers—one for rigid models and another for non-rigid models—allowing users to easily toggle visibility between them. A key feature of the system is its ability to switch between displaying rigid and nonrigid models using voice commands, providing an intuitive and hands-free method for users to alternate among different anatomical representations. The system also includes a visual indicator that displays the current connection status with the server, enhancing user awareness of the application’s state. The implementation is tailored to the specific requirements of the HoloLens^®^ 2 platform, utilizing Windows Universal Windows Platform (UWP) APIs for network communication and file handling, and incorporating error handling and logging to ensure robustness.^21^ After the models are successfully loaded and displayed, the client removes temporary files to manage storage efficiently. This system exemplifies a dynamic approach to loading and displaying complex 3D models in a mixed reality environment, particularly for medical applications such as surgical planning and training. The ability to switch between rigid and nonrigid models is especially valuable for comparing pre-operative and intra-operative anatomical states, highlighting the potential of mixed reality in enhancing visualization and interaction with medical imaging data.

### 2.7 Surface Target Evaluation

To evaluate the accuracy of the navigation system, surface targets were created on the liver phantom for a systematic comparison between ground truth and virtual target positions. Using a marker pen, 10 crosshairs were drawn on the liver phantom surface, representing the physical targets. These targets were then digitized and incorporated into the system for further analysis. The first step involved reconstructing the liver phantom surface using Adobe Substance 3D Sampler (Adobe Inc., San Jose, California, United States). A total of 55 photographs were captured from various angles to ensure comprehensive surface coverage. The software generated a 3D model of the liver phantom in a relative scale, which was then adjusted to real-world dimensions. A sphere with a known diameter of 2.5 cm was used as a reference to scale the reconstructed model accurately. After scaling, 3D crosshairs were created at each target position using Blender (Blender Foundation, Amsterdam, Netherlands). These crosshairs, measuring 5 mm per axis and modeled as small cylinders with a diameter of 0.5 mm, were designed for easy visualization in the virtual environment. The registration of the 3D crosshairs to the preoperative liver phantom model was performed using the wICP algorithm, as detailed earlier. These crosshairs, referred to as soft-tissue “targets” in this context, were used as virtual surface targets and were overlaid onto the physical liver phantom using the proposed framework. The accuracy of the system was assessed by measuring both the ground truth target positions and the corresponding virtual target positions as TRE.^22^

$$TRE(q)=|q_{v}-q_{p}|,$$ (2)

where $q_{p}$ is the coordinate of the physical target in tracker space and $q_{v}$ is the coordinate of the corresponding virtual target in tracker space. A stylus tracked by the Polaris Vega optical tracker (Northern Digital Inc., Waterloo, Ontario) was employed to precisely capture the coordinates of the physical crosshairs. Once the proposed system is applied and the virtual targets are aligned and displayed in HoloLens view, all virtual targets remain fixed relative to the reference tool. However, some virtual targets may appear beneath the actual phantom. To accurately measure their positions, the phantom can be temporarily moved to allow direct measurement as all virtual targets are fixed relative to the reference target. To thoroughly evaluate the system’s performance, this process was repeated across five deformation states of the liver phantom, resulting in a total of five datasets (10 soft-tissue targets per deformation state). The comparison between the ground truth and virtual target positions provided a detailed characterization of the system’s accuracy under different conditions. To ensure a comprehensive evaluation, three datasets were recorded for each deformation state: the positions of the physical crosshairs, the measured positions of the virtual crosshairs overlaid using RRM, and the measured positions of the virtual crosshairs overlaid using NRM. By comparing the TRE obtained from RRM and NRM, the analysis will highlight the superior accuracy achieved with NRM, underscoring its potential to enhance mixed reality applications.

## 3 Results

### 3.1 Data Acquisition Results

**Fig. 5:**
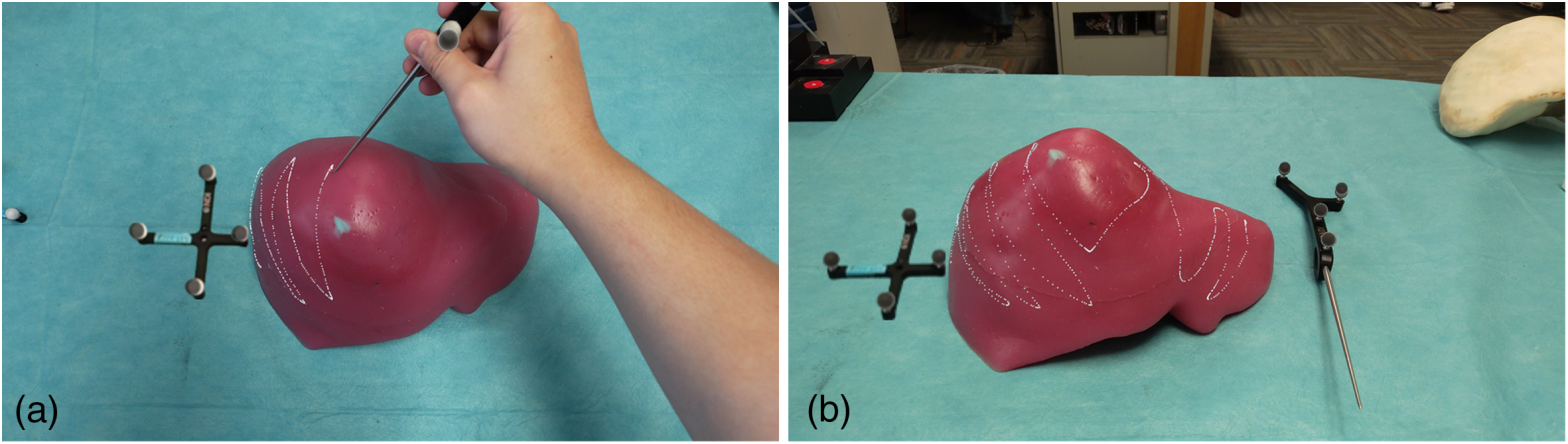
Surface data acquisition using the HoloLens^®^ 2 system showing (a) real-time point collection with a stylus tracked by HoloLens^®^ 2 and data being collected relative to global reference, and (b) visualization of the collected surface points overlaid on the liver phantom.

As noted, the ability to digitize the organ surface and other features is a critical capability for the guidance system. Figure 5 shows the surface data acquired by HoloLens® 2 used in the process. More specifically, the methodology in Sec. 2 is demonstrated using the onboard instrumentation of the HoloLens^®^ 2 to track a rigid body (stylus and reference) such that mock liver surface points are acquired as the liver surface is physically swabbed by the tracked stylus. Display capabilities built into the HoloLens SDK then enabled the mixed reality rendering of the acquired points into the video stream. To evaluate the fidelity of surface data acquired with the HoloLens^®^ 2–tracked stylus, we compared the reconstructed surface with a ground-truth model. Because the positions of four retro-reflective markers on the reference target were known in both HoloLens and image coordinate frames, the acquired point cloud could be transformed into image space, enabling a direct comparison with the ground-truth surface. The acquired points were resampled and fit to a smooth surface prior to evaluation. Quantitative assessment was performed using the modified Hausdorff distance (MHD), which yielded an average error of 2.6±1.2  mm. Although additional refinement is required to further improve accuracy, this level of agreement provides encouraging evidence of the HoloLens-based stylus system’s capability for reliable surface acquisition in its current form.

### 3.2 Virtual Model Stability Results

**Fig. 6:**
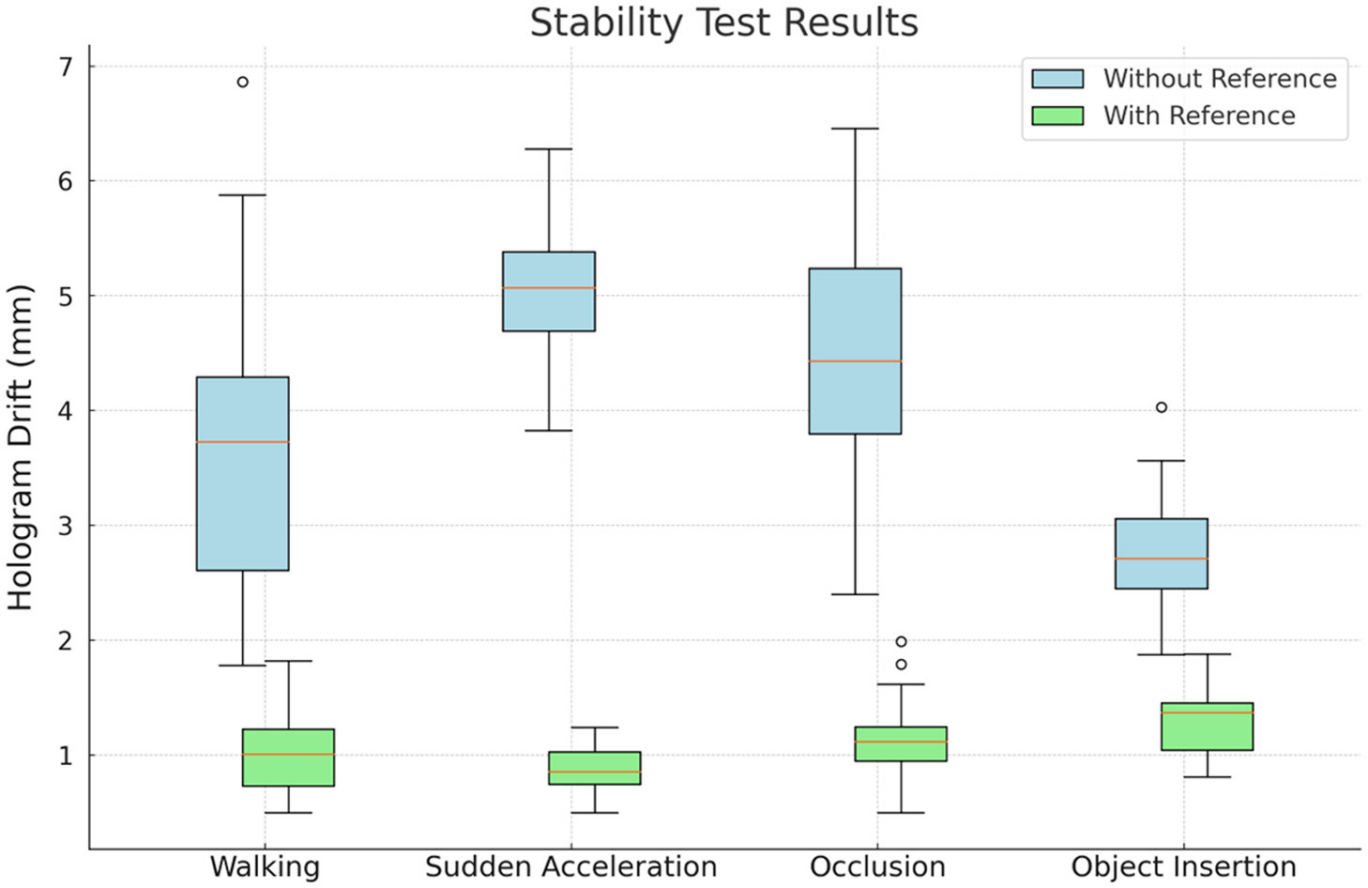
Hologram stability result without and with external reference for tasks: (a) walking, (b) sudden acceleration, (c) occlusion, and (d) object insertion.

A total of 40 hologram drift measurements were taken for each test type. The interquartile ranges and medians of these drift measurements by test type are displayed in Fig. 6. When it is based on HoloLens built-in SLAM function, the mean hologram drift of the walking, sudden acceleration, sensor occlusion, and object insertion trials is 3.7±1.1  mm (95% CI: 3.3 to 4.0), 5.1±0.5  mm (95% CI: 4.9 to 5.2), 4.5±1.0  mm (95% CI: 4.2 to 4.8), and 2.8±0.5  mm (95% CI: 2.6 to 2.9), respectively. When all four test types (160 trials) are considered together, the mean displacement is 4.0±1.2  mm (95% CI: 3.8 to 4.2). When there is an external reference target, the mean hologram drift of the walking, sudden acceleration, sensor occlusion, and object insertion trials is 1.0±0.4  mm (95% CI: 0.9 to 1.0), 0.9±0.2  mm (95% CI: 0.8 to 0.9), 1.1±0.3  mm (95% CI: 1.0 to 1.2), and 1.3±0.3  mm (95% CI: 1.2 to 1.4), respectively. When all four test types (160 trials) are considered together, the mean displacement is 1.1±0.3  mm (95% CI: 1.0 to 1.1). Therefore, the hologram stability is significantly improved by the external reference target (p=0.00048, paired t-test).

**Fig. 7:**
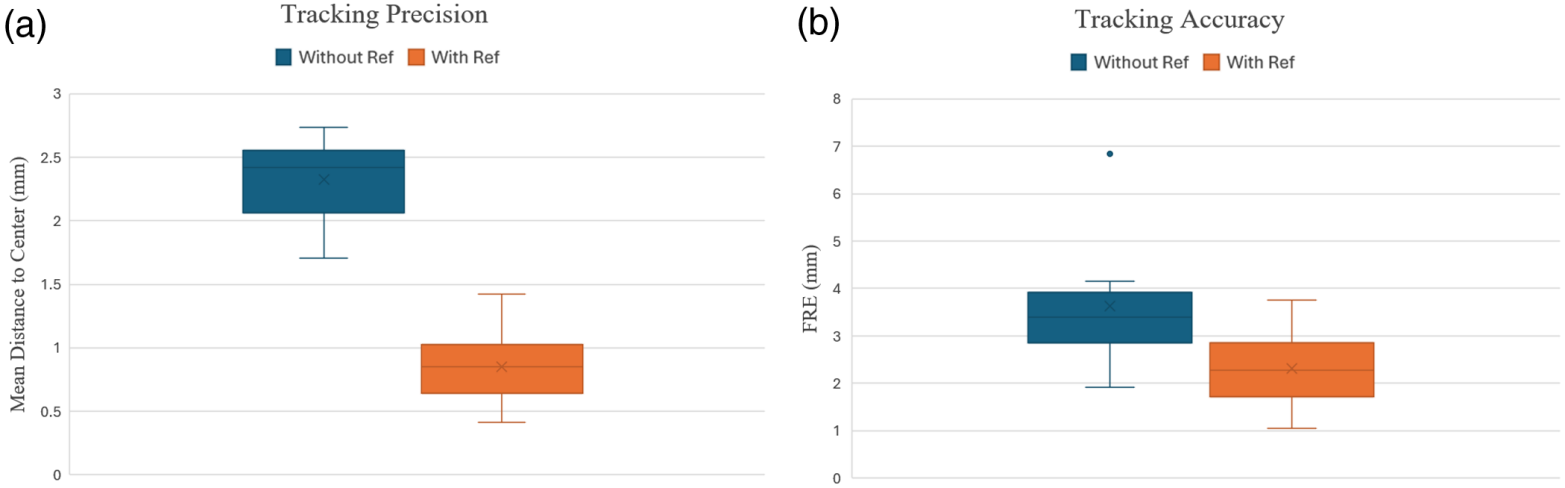
Comparison of HoloLens^®^ 2 tracking performance with and without reference tool showing (a) tracking precision measured by point cloud standard deviation and (b) tracking accuracy measured by fiducial registration error (FRE).

To analyze the HoloLens^®^ 2 stability further, a pivot test on a fixed point was performed as mentioned in Sec. 2.4. When the tracking is solely based on HoloLens^®^ 2 SLAM function, the mean standard deviation of the point clouds was found to be 2.3±0.3  mm, reflecting tracking precision at each measured point. By contrast, with the introduction of the external reference, the mean standard deviation of the point clouds was 0.8±0.3  mm, as shown in Fig. 7(a) (p=0.00028, paired t-test). After performing point registration, the mean distance between the identified centers of the point clouds and the known centers representing the tracking accuracy was 3.6±1.3  mm without the reference. By contrast, with the reference, the mean distance was 2.3±0.8  mm (p=0.0046, paired t-test). When considering tracking precision and accuracy, the system with the reference outperformed the system using HoloLens^®^ 2’s conventional environment stabilization methodology, i.e., SLAM.

### 3.3 Surface Target Alignment and Deformation Correction Results

**Fig. 8:**
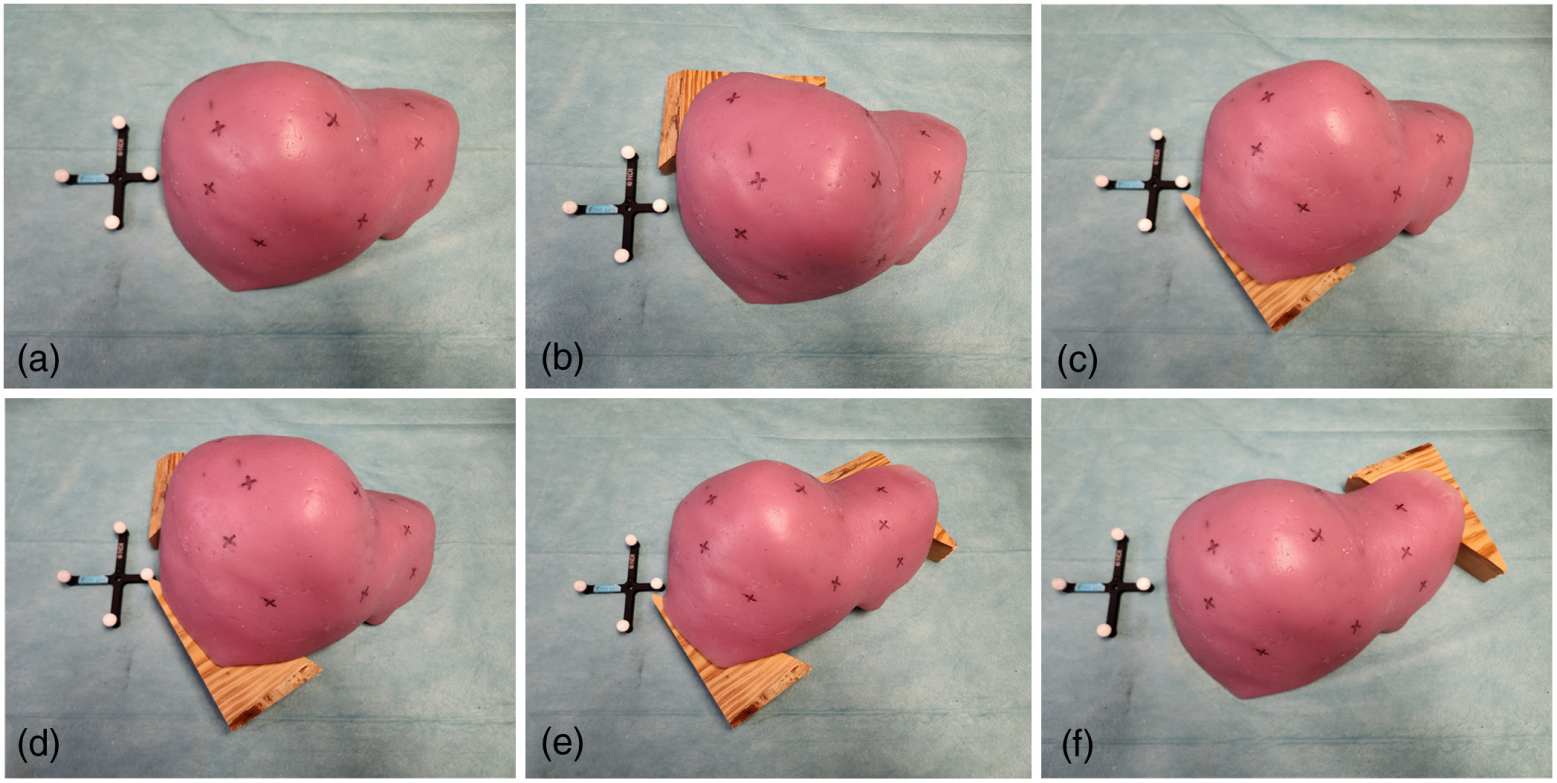
(a) Undeformed liver phantom. (b)–(f) Different states of liver phantom from deform 1 to 5.

**Fig. 9:**
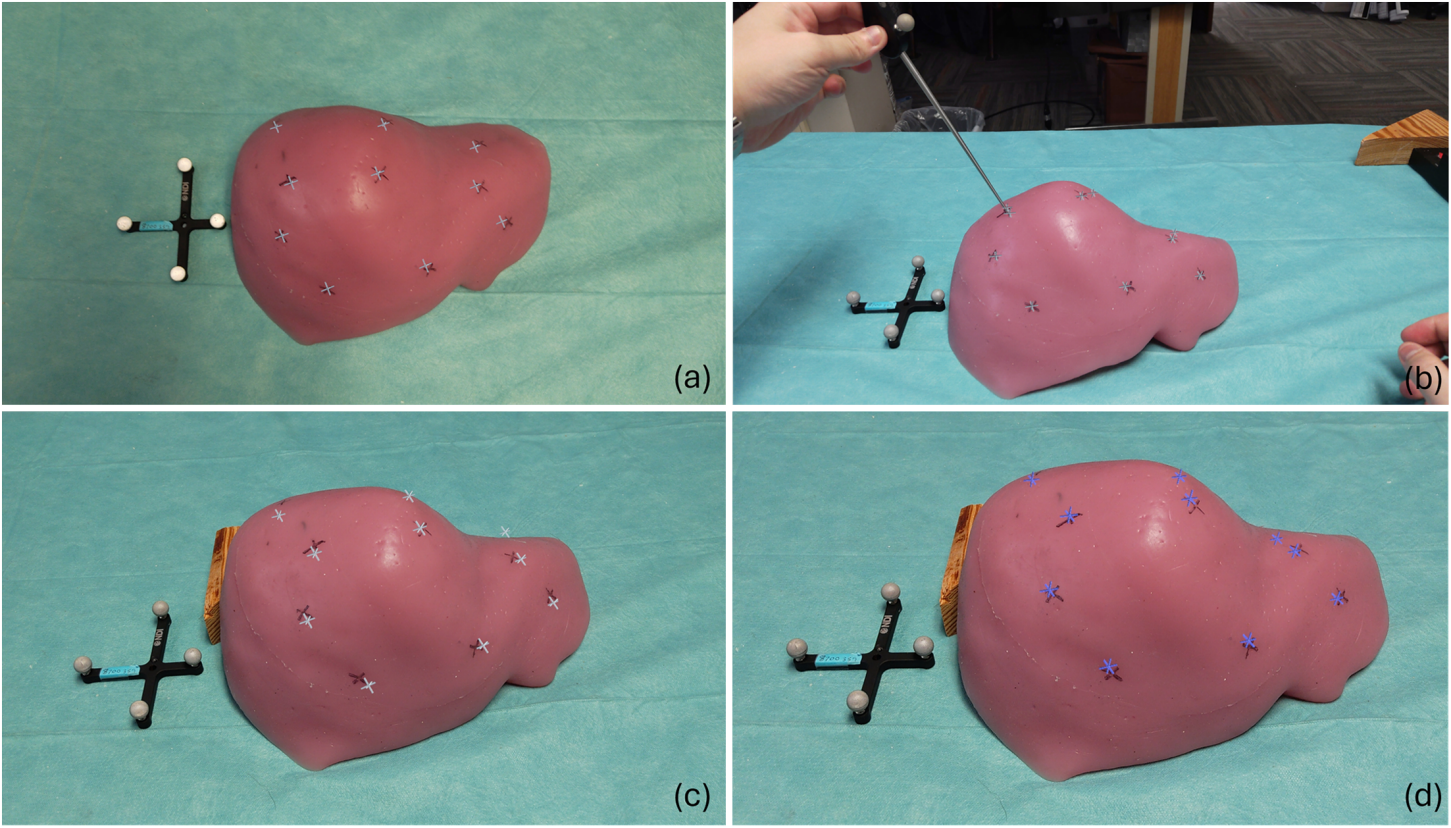
Surface target visualization and measurement. (a) Baseline configuration of the liver phantom without deformation. (b) Equivalent physical target points measured using an optically tracked stylus. (c) Deformation state 1 with virtual targets registered using the rigid registration method (RRM). (d) Deformation state 1 with virtual targets registered using the NRM, demonstrating improved alignment and compensation for soft tissue deformation.

The liver phantom was subjected to five distinct deformation states, as shown in Fig. 8, simulating surgical deformation due to mock intra-procedural mobilization or hepatic packing. Figure 9 is an example of the experimental setup and the visualization of virtual targets used in the performed experiments. Figures 9(a) and 9(b) show the baseline configuration without deformation, with Fig. 9(b) highlighting equivalent points measured by an optically tracked stylus. Figures 9(c) and 9(d) depict a deformed state, demonstrating the overlay of virtual targets registered using rigid and NRMs, respectively.

**Fig. 10:**
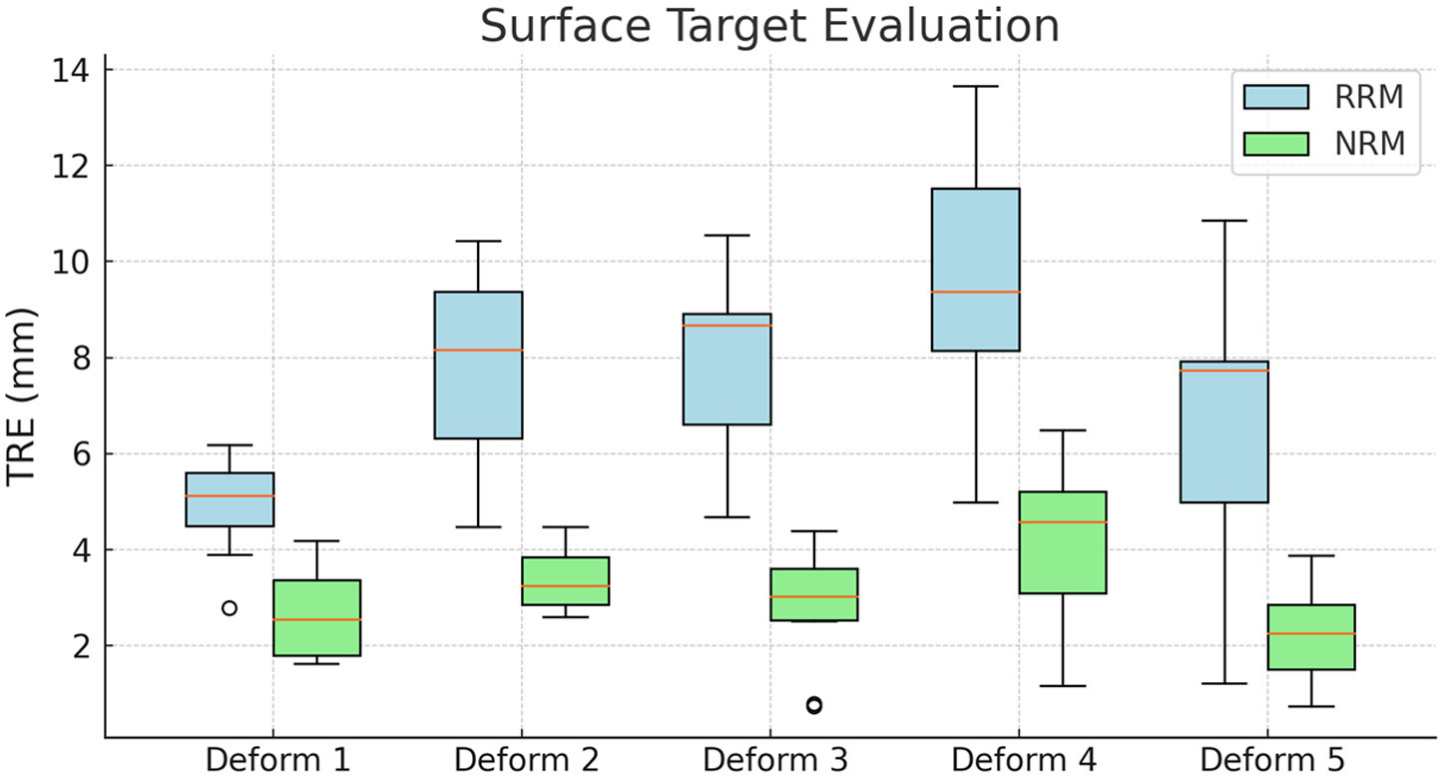
Visualization of surface target results for RRM and NRM under deformation conditions, highlighting the reduced TRE achieved with NRM compared with RRM across varying deformation states of the liver phantom.

The virtual surface targets were overlaid onto the deformed phantom surfaces using both rigid and NRMs. The average TRE across these cases was utilized to evaluate the overall system performance. To minimize target selection bias, target positions were evenly distributed across the phantom volume’s surface, as illustrated in Fig. 9(a). With careful inspection, the results in Fig. 9 demonstrate that the nonrigid method of registration [Fig. 9(d)] outperforms the rigid method [Fig. 9(c)] in the presence of deformation. Table 1 reports the TRE for each registration method across the entire testing suite and demonstrates a compensation ability ranging from 47% to 69% with rigid registration yielding an average TRE of 7.4±4.8  mm across the five deformation states, whereas the NRM achieved a significant improvement with an average TRE of 3.0±2.7  mm (p=0.00172, paired t-test). Figure 10 provides a graphical representation. These findings highlight the efficacy of the deformation correction approach and underscore its potential for clinical application in scenarios where precise alignment is critical.

**Table 1:** Surface target results.

TRE (mm)	RRM (95% CI)	NRM (95% CI)
Deformation 1	4.9 ± 0.9 (4.2 to 5.6)	2.6 ± 0.9 (2.0 to 3.3)
Deformation 2	7.8 ± 2.1 (6.3 to 9.2)	3.4 ± 0.6 (2.9 to 3.8)
Deformation 3	8.0 ± 2.0 (6.6 to 9.4)	2.8 ± 1.2 (1.9 to 3.7)
Deformation 4	9.8 ± 2.6 (7.9 to 11.6)	4.0 ± 1.9 (2.7 to 5.4)
Deformation 5	6.7 ± 2.7 (4.8 to 8.6)	2.3 ± 1.0 (1.6 to 3.0)
Mean	7.4 ± 4.8 (6.4 to 8.2)	3.0 ± 2.7 (2.6 to 3.4)

TRE results (reported as mean ± standard deviation, with 95% confidence intervals in parentheses) for RRM and NRM across five deformation states of a liver phantom, demonstrating improved accuracy with NRM in aligning virtual targets to the deformed surface.

This evaluation demonstrates the robustness of the MR-based navigation system in accurately overlaying virtual targets onto physical models, even under varying deformation conditions. Such accuracy is critical for clinical applications, where precise localization of anatomical structures is paramount for successful surgical navigation.

**Fig. 11:**
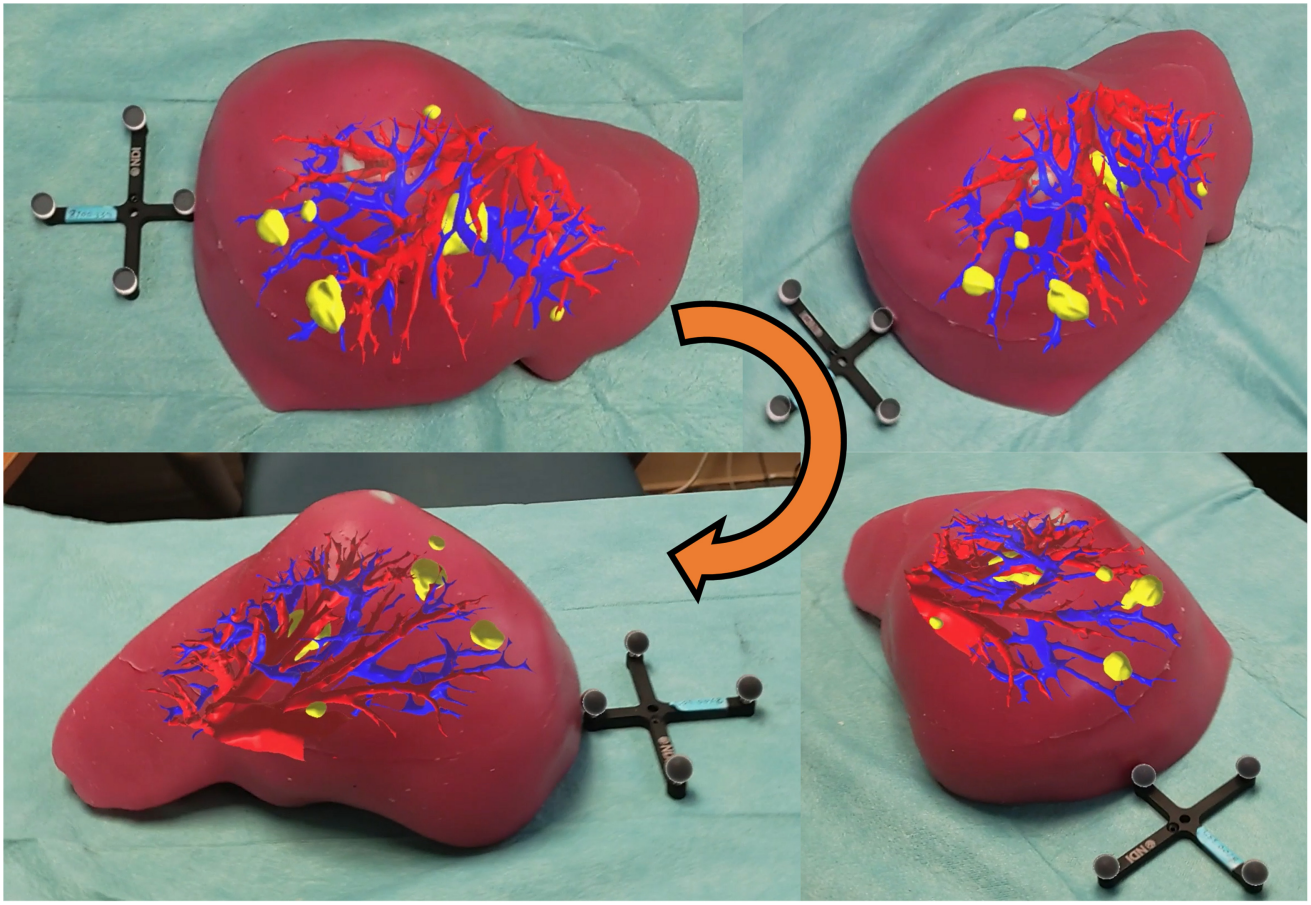
Visualization of subsurface liver anatomy in a liver phantom using the proposed surgical navigation system. The system accurately displays virtual models from various angles, demonstrating its stability and potential for use in liver surgery.

Figure 11 showcases the culmination of the work with the visualization of subsurface liver anatomy on a liver phantom using the surgical navigation system. The figure includes multiple views of the phantom, with the projected virtual models of liver subsurface vascular structures and lesions displayed in vivid colors. The models align with the physical phantom, demonstrating the system’s capability to maintain spatial stability and precise registration from different perspectives.

## 4 Discussion

The integration of an MR headset for liver surgery demonstrates a significant step forward in the realm of MR-enabled surgical navigation. Throughout this study, we evaluated several critical aspects of the system, including hologram stability, tracking accuracy, deformation correction, and the potential for future integration of new features. These findings provide important insights into the capabilities and limitations of MR systems in surgical applications while also identifying key areas for improvement and expansion. Although this study demonstrates implementation using the HoloLens^®^ 2 platform, the underlying framework’s architecture is device-agnostic, contingent primarily on depth sensor availability and accessible depth data streams. This generalized approach enables potential adaptation to alternative MR devices that meet these fundamental hardware requirements.

In terms of system performance, the workflow can be divided into three phases: tracking, deformation correction, and mixed-reality display. For tracking, no quantitative latency assessment was performed; however, preliminary observations indicated an approximate latency of 200 ms with a frame rate of 45 Hz, and ∼40  s was required to swab the liver surface for intraoperative data acquisition. Deformation correction was performed in two steps, with rigid registration requiring roughly 2 s and nonrigid LIBR correction ∼40  s. Once deformation-corrected models were generated, transferring the data from the PC to the HoloLens required an additional 30 s before visualization. Consequently, the entire pipeline from surface acquisition to holographic display took ∼2  min. It should be noted that the process does not support a continuous update rate as each new deformation event necessitates repeating the entire workflow. Future work will focus on accelerating these steps and optimizing the pipeline for improved efficiency.

One challenge identified during the evaluation was the mismatch between the field of view (FoV) of the AHAT camera, utilized for tracking, and the visualization range of the HoloLens^®^ 2. Specifically, the AHAT camera offers a wide FoV of 127  deg×127  deg, whereas the visualization range is significantly narrower at 43  deg×29  deg.^18^ This disparity can result in situations where tracked tools move out of the display’s visualization range, potentially disrupting the user’s spatial perception. In the specific context of our system, this issue becomes apparent when a surgeon attempts to observe the registered virtual liver anatomy. Although the aligned virtual sphere indicating the external reference tool may not be visible within the display’s narrower FoV, the AHAT camera’s wider FoV ensures that the reference tool remains actively tracked. It is critical to inform users that this discrepancy does not imply a loss of tracking, provided the tool remains within the AHAT camera’s tracking range. However, surgeons must remain aware of this limitation to avoid moving the tool beyond the AHAT camera’s coverage, which would result in an actual loss of tracking.

Another significant challenge pertains to virtual model occlusion, where virtual objects incorrectly obscure real-world tools. This issue is particularly critical for clinical applications, where the spatial hierarchy between virtual models and physical instruments must be accurately represented. Unlike the surface experiments herein, where occlusion had minimal impact, clinical use requires precise and consistent visualizations. Transparency adjustments, although initially appealing, can disrupt spatial relationships among virtual models themselves, exacerbating the problem. The root of this issue likely lies in the rendering materials and brightness levels. Advanced rendering techniques, such as dynamic material properties or occlusion-culling algorithms, should be explored to resolve this challenge without compromising the system’s usability. Another potential approach is to render a digital representation of real-world tools within the visualization layer, as seen in existing FDA-approved systems such as Medivis. However, this approach may require further innovation as not all tools may have predefined digital counterparts. Such improvements would enhance the visual fidelity and practical applicability of the system.

The evaluation of surface targets in this study provided valuable insights into the deformation correction capabilities of the system. By transitioning from rigid to NRMs, we demonstrated significant improvements in TRE, with nonrigid registration achieving an average TRE of 3.0±2.7  mm compared with 7.4±4.8  mm for rigid methods in the presence of organ deformations. In liver surgery, a commonly accepted benchmark is 5 mm, representing approximately half of the typical oncologic margin.^19^ Our results fall within this threshold, which highlights the effectiveness of incorporating biomechanical models to account for soft tissue deformations. However, surface target evaluation does not mimic the real clinical scenario, where all critical anatomical structures of interest—such as tumors and vessels—are located subsurface. Future studies should focus on subsurface target evaluations, using fiducials embedded within the liver phantom to assess the system’s accuracy in localizing internal structures. Such evaluations would provide a more comprehensive validation of our system, ensuring its readiness for complex clinical scenarios where subsurface precision is critical.

A promising future direction for this system is the integration of live ultrasound (US) imaging within the MR environment. Incorporating real-time MR US technology, as highlighted in recent studies,^23^ presents significant potential for enhancing intraoperative guidance. By tracking the US probe using retro-reflective markers and streaming live ultrasound images to the HoloLens^®^ 2, clinicians could visualize subsurface anatomical structures in real time, superimposed onto deformation-corrected 3D models. This integration would not only enrich the surgical field’s visualization but also serve as an additional layer of compensatory information, further enhancing the reliability of the system. Such an approach offers a more comprehensive understanding of the surgical field, facilitating improved decision-making. Moreover, this integration could also be leveraged to assess the accuracy of the system’s virtual object registration, drawing on our extensive experience with ultrasound-based evaluation methods.^24^ However, key challenges remain, including optimizing tracking frequency and achieving seamless alignment of ultrasound images with the MR environment. Addressing these challenges will be essential to fully realize the potential of ultrasound-MR integration, both within this system and in broader clinical workflows.

Although the current implementation has been validated in phantom studies, the system architecture readily facilitates integration into clinical workflows. For open surgical approaches, we propose incorporating a sterile tissue tracer equipped with reflective markers that attaches directly to the hepatic surface using standard surgical adhesives, establishing a stable coordinate reference. Although the utilization of active tracking from visual texture may be possible,^25^ the liver surface has limited textural features and lighting within the abdomen can vary. By designating this retroreflective marker as the world coordinate origin, the augmented reality environment maintains spatial registration with the physical anatomy throughout the procedure. This methodology parallels established techniques in neurosurgical navigation, where reference frames are rigidly secured to cranial structures while addressing the unique challenges of soft tissue deformation. Our system’s biomechanical modeling capabilities detect and compensate for volumetric shape changes, but the continuous tracking of this rigid reference remains essential for accommodating gross organ displacement, stabilizing the virtual environment, and ensuring accurate alignment of subsurface structures throughout the intervention. This approach provides the additional advantage of maintaining registration integrity despite respiratory-induced diaphragmatic motion affecting the operative field.

Although the current evaluation was performed in an open approach phantom setting, the end-to-end architecture we describe—external reference-anchored tracking, biomechanical deformation correction, and *in situ* MR visualization—is inherently modality-agnostic and can be transposed to laparoscopic liver surgery with only workflow-specific modifications. First, the reference tool can be miniaturized into sterilizable, trocar-compatible assemblies or integrated onto existing laparoscopic graspers, preserving the stable world-origin that mitigates hologram drift. Second, the tracked stylus would be redesigned as a slender, curved, sterilizable probe that can pass through the trocar; this laparoscopic stylus would still bear retro reflective markers and an encoded tip transform, allowing surgeons to sweep the hepatic surface and digitize salient features under pneumoperitoneum exactly as described in Sec. 2.5. The point clouds thus acquired feed directly into the same rigid + LIBR non rigid registration pipeline, with a deformation basis that can already accommodate insufflation induced shape change. Finally, the server–client streaming mechanism already supports real-time model updates; coupling it with synchronized laparoscope video would allow surgeons to toggle between conventional monitor-based views and holographic overlays without cognitive context switching. Collectively, these adaptations position the proposed MR platform as a practical adjunct for laparoscopic liver resection or ablation, promising the same accuracy gains demonstrated here while respecting the spatial and ergonomic constraints of minimally invasive surgery. Although these points illustrate the system’s potential for laparoscopic surgery, effectively adapting it in practice will necessitate a considerable amount of additional work.

These findings and proposed enhancements underscore the transformative potential of MR-enabled surgical navigation systems. By addressing current limitations and incorporating advanced features such as ultrasound imaging, the HoloLens® 2-based system can evolve into a more robust and versatile tool for surgical precision. Future work should prioritize comprehensive validation studies in real-world surgical settings, evaluating the system’s performance across diverse scenarios and patient anatomies. In addition, considerations such as ergonomic design, user training, and system robustness must be integrated into the development process to ensure seamless adoption by surgical teams. Ultimately, these advancements will contribute to the broader goal of improving surgical outcomes and setting a new standard for precision and efficacy in liver surgery and beyond.

## 5 Conclusion

The development and evaluation of the MR-based surgical navigation system for liver surgery presented in this study demonstrate significant advancements in MR-enabled surgical guidance. By integrating robust tool tracking, deformation correction, and dynamic 3D visualization, the system addresses critical limitations of conventional approaches, including inaccuracies due to intraoperative tissue deformation and challenges with hologram stability. Experimental results show substantial improvements in precision, with NRMs achieving an average TRE of 3.0±2.7  mm compared with 7.4±4.8  mm for rigid registration. The integration of an external reference tool effectively mitigated hologram drift, ensuring spatial stability even under dynamic surgical conditions, and enhancing the reliability of MR overlays. These findings validate the feasibility of employing mixed reality for precise intraoperative guidance while highlighting opportunities for further refinement, including the evaluation of subsurface targets and integration with live imaging modalities such as ultrasound. This study underscores the transformative potential of MR-based navigation systems, marking a significant step toward their adoption in complex surgical procedures. Future research should focus on comprehensive *in vivo* validation and optimizing the system’s adaptability to diverse surgical scenarios, advancing the precision and efficacy of modern surgical interventions.

## Data Availability

The code and data used in this study were developed by the authors and are not publicly available due to intellectual property and institutional restrictions.
